# Functional soil microbes drive soil phosphorus fractions in response to nitrogen addition across aggregate levels

**DOI:** 10.3389/fmicb.2025.1671863

**Published:** 2025-10-21

**Authors:** Jiaxin Hu, Haiying Cui, Mingcai Fan, Min Liu, Shanling Wang, Xiuping Li, Xia Peng, Fengxue Shi, Wenzheng Song, Wei Sun

**Affiliations:** ^1^Key Laboratory of Vegetation Ecology, Institute of Grassland Science, School of Life Science, Northeast Normal University, Ministry of Education, Jilin Songnen Grassland Ecosystem National Observation and Research Station, Changchun, China; ^2^Jilin Provincial Natural History Museum, Northeast Normal University, Changchun, China; ^3^College of Tourism, Resources and Environment, Zaozhuang University, Zaozhuang, China

**Keywords:** soil *phoD*-harboring bacteria, phosphorus fractions, soil aggregate, nitrogen addition, alkaline phosphatase activity

## Abstract

Phosphorus (P) is one of the most important limiting nutrients for plant productivity in terrestrial ecosystems. As key drivers of P cycling processes, changes in soil microbial diversity and community structure can influence soil P cycling and availability. Nitrogen (N) deposition, as a global change factor, profoundly alters soil P cycling; yet how soil P fractions respond to N addition across multiple gradients, and the potential mechanisms driven by plant, microbial, and soil properties at the soil aggregate level, remains unclear. In this study, we conducted a seven-year, long-term field experiment to investigate the response patterns of soil labile and non-labile P fractions to N addition at the four gradient levels (0, 5, 10, and 20 g N m^−2^ y^−1^) in macroaggregates and microaggregates in a meadow steppe in Northeast China. We found that N addition reduced the content of soil non-labile P in macroaggregates, but increased all P fractions in microaggregates. Soil functional microbes play different roles in driving soil P fractions. Soil labile and non-labile P fractions were mainly controlled by the diversity and gene abundance of soil *phoD*-harboring bacteria, and plant and soil properties in macroaggregates, but by soil microbial stoichiometry in microaggregates. Moreover, N addition indirectly regulated P fractions by altering microbial functional traits, rather than directly by the changes of soil nutrient availability. Our results demonstrate that the mechanisms by which soil functional microbes and microbial stoichiometry regulate soil P fractions and transformation vary among soil aggregates. This study provides new insights into the crucial role of soil functional microbes in improving P supply by accelerating the process of soil P fractions under global change scenarios. To enhance sustainable grassland development in the changing world, we need to prioritize the leveraging of soil aggregate-mediated processes in grasslands.

## Introduction

As an essential macronutrient, phosphorus (P) regulates the key biochemical and physiological processes that are critical to plant growth in terrestrial ecosystems (Vitousek et al., 2010). It ranks as the second-most limiting nutrient for plant growth after nitrogen (N) (Vitousek et al., 2010). This limitation arises primarily from the slow weathering rates of P-containing source rocks, which restrict the natural release of bioavailable soil P (Walker and Syers, 1976), leaching, and adsorption processes (McGill and Cole, 1981; Elser, 2012). Soil P fractions enhance P availability under P-deficient conditions, meeting the increased P demand of plants and microbes as the key components of the soil P pool. However, knowledge gaps remain regarding how aggregate-scale P dynamics interact with environmental changes, limiting the development of effective grassland management strategies under global change.

Soil aggregates, as fundamental structural units, mediate nutrient distribution through variations in composition, stability, and microbial characteristics (Su et al., 2021; Ma et al., 2025). Strong P adsorption and precipitation within aggregates drive size-dependent mechanisms of P retention and supply (Tiessen et al., 1984; Wu et al., 2021; Zhao et al., 2021). Recent studies also reveal that N enrichment alters aggregate-scale P distribution, with significant declines in soil labile P in macroaggregates under chronic N addition, despite the stability of microaggregates (MAs) (Cui et al., 2025). MAs show distinct organic P accumulation influenced by soil fertility. Organic P in macroaggregates is more easily mineralized to release plant-available P, whereas P bound in microaggregates is stabilized and preserved as a key soil P pool (Tian et al., 2022; Wan et al., 2025). Contrasting findings exist: Jastrow (1996) demonstrated higher organic carbon in large macroaggregates (LMAs) than in MAs. Further empirical evidence has demonstrated that LMAs contain greater amounts of carbon (C), nitrogen (N), and labile organic matter than small macroaggregates (SMAs). Conversely, one research team observed declining C, N, and P concentrations, but increasing stocks with larger aggregate sizes (Yan et al., 2023; Mbibueh et al., 2024). However, the mechanisms driving soil P fraction responses to global changes in grassland ecosystems remain poorly understood.

Intensive agricultural and industrial activities have profoundly altered global biogeochemical cycles, with atmospheric N deposition emerging as a primary driver of environmental change (Yang et al., 2024). Elevated N deposition modifies aggregate distribution and subsequent nutrient availability (Marklein and Houlton, 2012; Bai et al., 2023; Cheng et al., 2024). The biological significance of P fractions, categorized by stability and responsiveness to N inputs, varies significantly, reflecting their distinct roles in ecosystem functioning (Yang et al., 2021). Previous studies show that N enrichment can reduce soil P availability by stimulating root and microbial phosphatase secretion. This enhances plant P acquisition and expands plant P pools to maintain N–P stoichiometric balance, ultimately depleting soil P reserves (Menge and Field, 2007; Hou et al., 2018; Margalef et al., 2021). However, other evidence suggests that N-induced soil acidification can mobilize non-labile P, increasing soil P availability or enhancing plant P resorption efficiency. This alleviates P limitation in grasslands and raises both total and bioavailable P concentrations (Zhang et al., 2014). These paradoxical findings highlight the complexity of N–P interactions in terrestrial ecosystems and the need for deeper investigation (Xin et al., 2025; Zhang T. et al., 2023). The dynamics of soil P fractions within aggregates under N deposition, and their underlying mechanisms, remain poorly understood. This limits our ability to predict how global change will reshape soil P cycling and affect grassland productivity.

Soil microbes play a pivotal role in regulating P cycling and fractions, particularly under N deposition. Three alkaline phosphatase gene families—*phoD*, phoA, and phoX—have been identified in soils (Jiang et al., 2024; Wang et al., 2024; Pan et al., 2024). Among them, the bacterial *phoD* gene, broadly distributed in terrestrial and marine environments, encodes alkaline phosphatase (ALP), an enzyme responsible for mineralizing soil organic P and widely recognized as a key biomarker of ALP activity (Wang et al., 2020; Chrost et al., 1984). The diversity and abundance of *phoD*-harboring bacteria are strongly shaped by nutrient input rates, making them crucial indicators of soil P transformation potential (Sakurai et al., 2008; Cui et al., 2025). Long-term enrichment experiments further reveal that *phoD* gene abundance and microbial phosphatase activity vary with aggregate size, with smaller aggregates in acidic soils often showing higher activity (Sun et al., 2023a; Yang et al., 2023). Together, these findings underscore the central role of microbes in controlling P cycling and availability across soil aggregates, highlighting the need to clarify how N enrichment shapes the structure and function of *phoD*-harboring microbial communities at different aggregate levels.

Microbial stoichiometric ratios critically regulate P availability by determining microbial nutrient demands (Cui et al., 2021; Yu et al., 2024). Recent evidence demonstrates that the stoichiometry of soil microbial biomass (C:N:P) regulates the partitioning of phosphorus (P) into microbial biomass P (MBP) (Chen et al., 2019, 2024b). However, N deposition offers limited insight into how stoichiometry and microbial diversity shape P fractions within aggregates (Tamiru et al., 2024; Pang et al., 2024). Using a long-term nutrient experiment in a northeastern Chinese meadow steppe, we aim to assess the effects of gradient N additions on soil P fractions in macro- and microaggregates, with a focus on microbially mediated pathways. We hypothesize that: (1) N addition differentially influences soil labile and non-labile P fractions depending on aggregate size, with N addition in MAs increasing both soil labile and non-labile P contents. (2) The microbial mechanisms regulating the contents of soil labile and non-labile P fractions vary among aggregate size classes. In macroaggregates, the *phoD* gene abundance and diversity play a major role, while in MA, stoichiometry is the primary influencing factor. Integrating these findings will enhance our understanding of how microbial, plant, and soil interactions drive P dynamics in the context of global change, providing valuable insights for sustainable grassland management.

## Materials and methods

### Study site

The study area was conducted at the Jilin Songnen Grassland Ecosystem National Observation and Research Station, located in Jilin Province, China (44 °45′N, 123 °45′E). Since 2015 and up to now, these experiment plots in the meadow steppe have been subjected to multiple N addition treatments. Statistical data from 1953 to 2021 indicate that the area has an average yearly temperature of 6.4 °C and a mean annual rainfall of 445 mm. The vegetation growing period typically extends from May to September during this timeframe (Zhong et al., 2019). It is a typical farming-pastoral ecotone with a rich diversity of vegetation. The dominant species in the grassland community include *Leymus chinensis, Phragmites australis*, and *Puccinellia tenuiflora*, among others, which grow scattered throughout the area. In some parts of this meadow steppe, there is salinization. In areas without salinization, the soil is primarily chernozem, which exhibits a pH value of 8.0–10.0, indicating strong alkalinity.

### Experimental design

In 2010, a 2-hectare (100 × 200 m) permanent experimental exclosure was established using perimeter fencing. The exclosure was designed to systematically exclude anthropogenic disturbances, particularly livestock grazing, in order to isolate biotic responses from human activity and establish baseline conditions for controlled manipulative experiments. In May 2015, we established four experimental blocks under a randomized complete block design (RCBD), each holding four permanent 5 × 5 m^2^ plots systematically assigned to distinct treatments. Between 2015 and 2021, five rounds of N addition treatments were conducted from May to September. We applied four N treatments (designated N0–N20) at rates of 0, 5, 10, and 20 g N m^−2^ yr^−1^.

### Characterization of microbial, soil, and plant properties

In late July 2021, we assessed plant species richness using three 0.25 m^2^ permanent plots per experimental unit. Within each plot, a representative quadrat was randomly selected, and all above-ground plants were clipped close to the soil surface with scissors. To determine aboveground plant biomass (AGB), we oven-dried freshly cut samples at 105 °C for 2 h, then maintained them at 65 °C until weight stabilization. Within the same quadrat, 10-cm soil samples were obtained via root coring to ensure sampling consistency. The same procedures were adopted to obtain the belowground plant biomass (BGB).

We quantified total plant total P using the molybdenum blue colorimetric method, which is based on phosphorus–molybdenum complex formation (Van Veldhoven and Mannaerts, 1987). We digested 0.2 g of dominant *Leymus chinensis* tissue using 4 mL of H_2_SO4 and 6 mL of H_2_O_2_ at 375 °C for 3 h in a digestion oven. The resulting samples were then ready for analysis using the analyzer.

In late July 2021, soil sampling was conducted in each experimental plot employing a five-point sampling strategy. Soil samples were collected once in each plot. To avoid contamination, we chose to collect soil samples on the same day. We collected soil samples using a 2.5-cm-diameter auger to ensure spatial coverage representativeness, then immediately homogenized and sieved five soil subsamples through a 5-mm mesh to remove coarse material before laboratory transport. Subsequently, a sieve nest featuring 2,000- and 250-μm meshes was utilized for the soil samples. This sieve nest was connected to a Retsch AS200 control device (Retsch Technology, Düsseldorf, Germany). Soil samples were mechanically shaken for 2 min at an amplitude of 1.5 mm, resulting in three aggregate fractions: large macroaggregates (LMA, >2 mm), small macroaggregates (SMA, 0.25–2.00 mm), and microaggregates (MA, < 0.25 mm) (Yuan et al., 2021; Wang et al., 2010).

Soil moisture content (SMC, %) was determined based on the weight loss of fresh soils following oven-drying at 105 °C over a 24-h period. Soil organic carbon was quantified by removing total inorganic carbon with 1 M HCl from 1 g of dry soil, followed by an analysis on a Vario organic C analyzer (Elementar, Hanau, Germany). We extracted soil available nitrogen with 2 mol/L KCl, filtered the solution, and quantified concentrations using a FUTURA flow analyzer (FUTURA, Alliance-AMS, Frépillon, France) (Hart et al., 1994). All equipment calibration was performed using reference standards and a standard curve method. A method described by Jenkinson et al. (2004) involved determining microbial biomass C (MBC) through chloroform fumigation–extraction, with microbial biomass (MBN) and microbial biomass P (MBP) calculated from the differences among samples that were fumigated vs. those that were not fumigated. For MBC and MBN analyses, 5 g of fresh soil underwent 24-h chloroform fumigation, followed by extraction with 0.5 M potassium sulfate at a 1:10 soil-to-solution ratio (w/v) via 30-min shaking. Non-fumigated samples were included as control replicates. Dissolved organic C and available N in the extracts were concurrently quantified using a Vario organic C analyzer (Vario TOC, Elementar, Hanau, Germany). Final MBC and MBN values were then derived by multiplying the fumigated–unfumigated differences by a correction factor of 0.45 (Vance et al., 1987; Brookes et al., 1985). To measure MBP using the same fumigation method, 2.5 g of fresh soil was extracted with 0.5 M sodium bicarbonate solution at a 1:20 soil-to-solution ratio (w/v). P concentration was determined with an automated discrete analyzer (SmartChem 450, Alliance-AMS, Rome, Italy). Following the chloroform fumigation–extraction method, the MBP content was derived by multiplying the P concentration difference between fumigated and unfumigated samples by a 0.4 conversion factor (Kouno et al., 1995).

In this study, the ALP activity in soils with different aggregate sizes was assayed in 96-well microplates following the method described by Bell et al. (2013). The substrate used in this study was 4-methylumbelliferyl phosphate (MUB-P), which releases 4-methylumbelliferone (MUB) upon enzymatic hydrolysis. MUB can be excited at 365 nm and emits fluorescence at 460 nm, with fluorescence intensity indicating enzyme activity. Approximately 1 g of freeze-dried soil was weighed and placed into a 150-mL Erlenmeyer flask, followed by the addition of 100 mL of 50 mM sodium acetate buffer (pH 9.0). The mixture was shaken at 200 rpm for 30 min to obtain a soil suspension. A 200 μl aliquot of the suspension was then transferred into a black 96-well microplate, to which buffer solution, standard solution, and substrate solution were sequentially added. To simulate average soil temperature conditions, the assay plate was dark-incubated at 25 °C for 3 h, and measurements were conducted using a fluorescence microplate reader (TECAN Infinite F200, Tecan Group Ltd., Männedorf, Switzerland) (Trivedi et al., 2016). We quantified fluorescence with optical parameters set at 365 nm and 460 nm. To express enzyme activity in standardized units, measurements were first calibrated against quench controls and ultimately normalized as nmol·h^−1^·g^−1^ dry soil (nanomoles per hour per gram of dry soil). Following a modified protocol from (Frostegård and Bååth 1996), soil phospholipid fatty acids (PLFA) were extracted from 8 g of lyophilized soil using a phosphate-buffered solution (pH 7.4 ± 0.5), chloroform, and methanol. Subsequent fractionation of non-target lipids was achieved through solid-phase extraction chromatography. Fatty acid methyl esters were generated through lipid methylation using a DB-5 capillary column. Subsequent analysis was conducted via gas chromatography with flame ionization detection and capillary gas chromatography–mass spectrometry.

Soil microbial biomarkers were assigned according to established conventions: We identified Gram-positive bacteria (G^+^) using cy17:0, i17:0, i16:0, i15:0, a15:0, and a17:0, whereas Gram-negative bacteria (G^−^) were characterized by 18:1ω6c, 16:1ω7c, 16:1ω6c, and cy19:0 and actinomycetes using 10Me18:0 and 10Me17:0. Total bacterial biomarkers included i19:0, 18:1ω5, 18:1ω7, a17:0, 17:0, i17:0, 16:1ω7, i16:0, 16:1ω9, a15:0, i15:0, 15:0. Fungal communities were characterized by the biomarkers 18:1ω9c and 18:2ω6,9, while arbuscular mycorrhizal fungi (AMF) were identified by the lipid marker 16:1ω5 (Olsson et al., 1995; Frostegård et al., 1991; Frostegård and Bååth, 1996; Bååth and Anderson, 2003). Biomass quantification of respective microbial groups (total bacteria, actinomycetes, G^+^, G^−^, fungi, and AMF) was performed using standardized conversion factors as previously described, with resultant values subjected to multivariate statistical analysis.

Soil P fractions were characterized through an established sequential fractionation protocol adapted from Hedley and Tiessen methodologies (Tiessen and Moir, 1993; Hedley and Chauhan, 1982). The extracted fractions were systematically categorized into nine operationally defined pools: NaHCO3-Pi, HCl-Pi, NaHCO3-Po, HCl-Po, resin-P, NaOH-Pi, NaOH-Po, NaOHus-Pi, and NaOHus-Po (Crews et al., 1995; Pätzold et al., 2013). Total P represented the summation of all fraction pools. Based on established biogeochemical behavior and plant bioavailability, these fractions were grouped into two functional classes: soil labile P (i.e., NaHCO3-Pi, NaOH-Pi, NaOH-Po, NaHCO3-Po, and resin-P) and soil non-labile P (i.e., NaOHus-Pi, HCl-Pi, HCl-Po, and NaOHus-Po) (Fan et al., 2019).

### Measurement of soil *phoD*-harboring bacteria

Soil genomic DNA was extracted from 0.5 g fresh soil using commercial kits (Omega or MOBIO, USA), and DNA concentration and purity were verified with a NanoDrop spectrophotometer and agarose gel electrophoresis. The *phoD* gene abundance was quantified by qPCR with primers ALPS-F730 (5′-CAGTGGGACGACCACGAGGT-3′) and ALPS-1101 (5′-GAGGCCGATCGGCATGTCG-3′) (Sakurai et al., 2008). Each reaction contained diluted DNA template, SYBR Green PCR premix, and primers, and was run under the following program: The amplification program was performed in a thermal cycler with an initial denaturation at 98 °C for 5 min, followed by 35 cycles of denaturation at 98 °C for 30 s, annealing at 59 °C for 30 s, extension at 72 °C for 45 s, and final extension at 72 °C for 5 min (Huang et al., 2017). A plasmid carrying the *phoD* gene was cloned, sequenced, and serially diluted to generate standard curves for quantification.

For community analysis, *phoD*-harboring bacterial sequences were obtained by PCR amplification with the same primer pair, followed by purification and library construction. Sequencing was conducted on an Illumina MiSeq platform (Personal Biotechnology, Shanghai, China) using the MiSeq Reagent Kit v3. Raw sequences were filtered with TrimGalore, and chimeras were removed with UCHIME. Protein-coding regions were predicted and aligned against the FunGene *phoD* reference database using FrameBot. Sequences with premature stop codons, misalignments, or < 80% identity to validated *phoD* orthologs were discarded. To ensure comparability, all samples were rarefied to the same sequencing depth.

Operational taxonomic units (OTUs) were defined at 97% sequence similarity using UCLUST (Edgar, 2010). Alpha diversity indices (e.g., Shannon index) and community composition were then calculated to evaluate differences in *phoD* bacterial communities across aggregate fractions.

### Statistical analyses

We performed two-way ANOVA across aggregate size classes to examine:(1) N addition and aggregate effects on total, labile, and non-labile P; (2) soil properties (organic C, SMC, pH, available N); (3) plant parameters (total P, AGB, BGB, plant richness); (4) microbial metrics (MBC, MBP, MBN, PLFA, ALP activity, *phoD* diversity, *phoD* gene abundance). One-way ANOVA with Tukey's HSD tests compared nitrogen gradients. After confirming data normality via Shapiro–Wilk testing, we applied root or log transformations where appropriate. Spearman correlation and Mantel test analyses, combined with heatmaps, were used to visualize association networks among soil P fractions, soil properties, plant traits, and microbial characteristics within each aggregate class. Random forest modeling and redundancy analysis (RDA) were conducted to identify the primary drivers of soil P fractions per aggregate class. Partial least squares path modeling (PLS-PM) was subsequently applied to determine how plant traits, soil properties, and microbial metrics directly and indirectly regulate labile and non-labile P dynamics across distinct aggregate classes. Statistical analyses were conducted in R version 4.0.5 (http://cran.r-project.org/).

## Results

### Among soil aggregates, responses of soil P fractions to N addition

**Figure 1:**
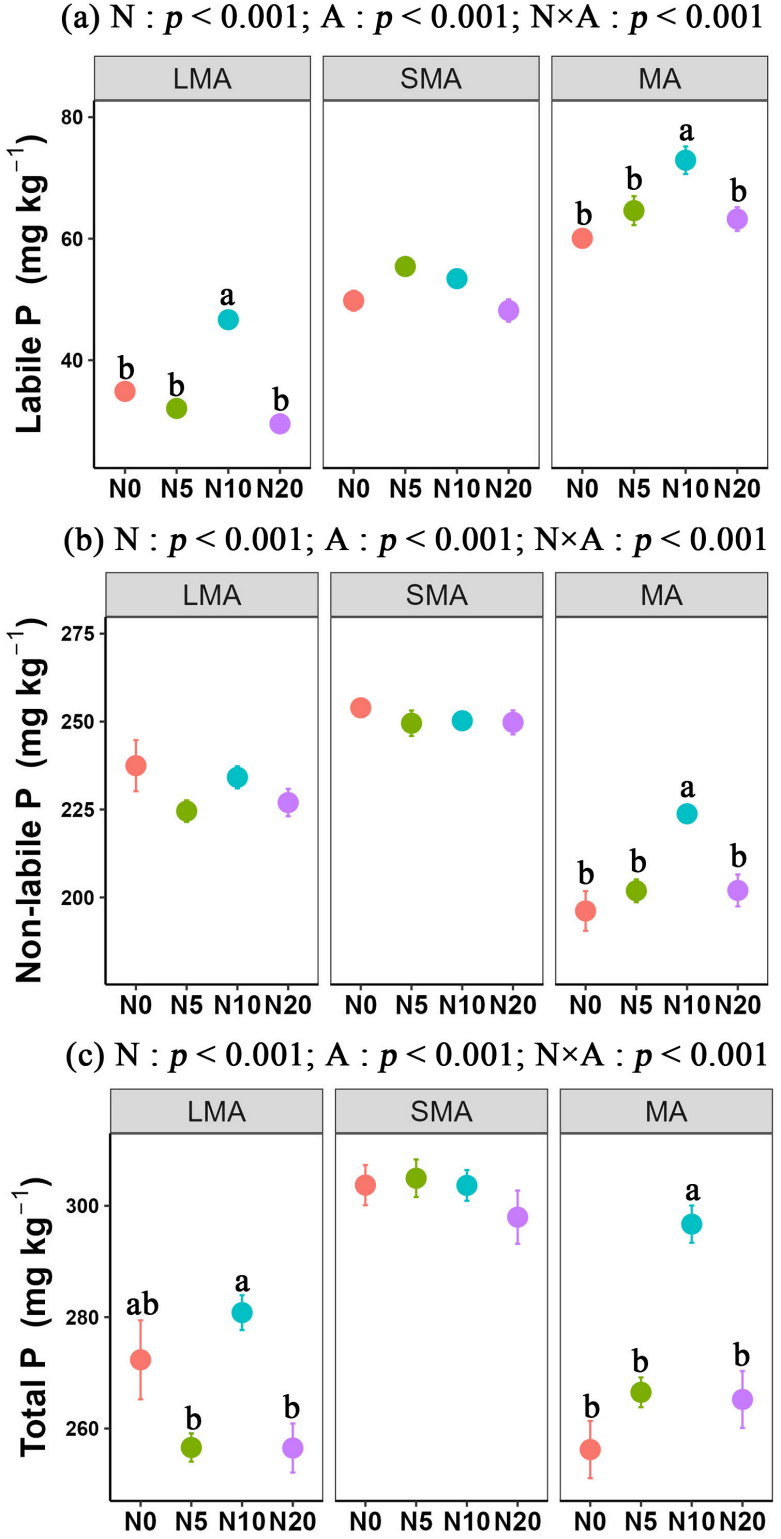
Responses of soil P fractions to N addition at three aggregate-size. The statistical results are two-way ANOVA testing for effects of N addition (N) and aggregate (A). Different lower-case letters indicate significant differences (one-way ANOVA, LSD's HSD, *p* < 0.05) among N addition in the same aggregate. Large microaggregates (LMA, >2 mm), Small microaggregates (SMA, 0.252 mm) and microaggregates (MA, <0.25 mm). Labile P (i.e., resin-P, NaHCO_3_-Pi, NaHCO_3_-Po, NaOH-Pi, NaOH-Po), Non-Labile P (i.e., NaOHus-Pi, NaOHus-Po, HCl-Pi, HCl-Po).

For soil labile P, the content sequence followed the order SMA> MA> LMA, while non-labile P and total P followed an order of SMA> LMA> MA. Our results also showed that N additions significantly altered soil P fractions across different soil types. In LMA, N addition led to a clear decline in P fractions. In LMA, labile P decreased by 15.3% with N addition at 20 g N m^−2^ yr^−1^ (from 34.87 ± 0.73 mg kg^−1^ with no N added to 29.53 ± 1.36 mg kg^−1^). Similarly, non-labile P declined by 4.4% (from 237.47 ± 7.27 mg kg^−1^ to 226.97 ± 3.89 mg kg^−1^). All these reductions were statistically significant. In contrast, N addition significantly promoted P accumulation in MA. Labile P increased by 21.4% at 10 g N m^−2^ yr^−1^ (from 60.06 ± 1.31 mg kg^−1^ with no N added to 72.92 ± 2.27 mg kg^−1^). Non-labile P increased by 14.1% (from 196.17 ± 5.64 mg kg^−1^ to 223.78 ± 2.59 mg kg^−1^). These increases were significant. However, in SMA, N additions did not significantly affect P fractions (Figures 1a–c).

### Responses of microbial, soil, and plant properties to N addition

N addition was found to significantly enhance both aboveground (AGB) and belowground biomass (BGB) (Supplementary Figures S1a, b) of plants. It is worth highlighting that both total plant P and species richness dropped significantly due to N addition (Supplementary Figures S1c, d). N addition significantly increased available N across all aggregate size classes (Supplementary Figure S2a). In LMA and SMA, soil pH increased at the N5 level, while in MA, soil pH increased under both N5 and N10 treatments. N addition at other levels led to a significant decrease in soil pH (Supplementary Figure S2b). In LMA and MA, N addition significantly decreased total organic C, whereas N10 significantly increased organic C in SMA (Supplementary Figure S2c). N addition reduced SMC across all aggregate classes, though not significantly.

N addition had distinct effects on soil microbial biomass, functional diversity, and stoichiometry among soil aggregate fractions. In LMA and SMA, soil MBC and MBN exhibited a biphasic response to increasing N inputs, decreasing initially before recovering, although MBN failed to rebound in both fractions (Supplementary Figures S3a, b). Soil MBP was only significantly promoted at the highest N level (N20) in LMA and SMA (Supplementary Figure S3c). Concurrently, soil *phoD* Shannon diversity, *phoD* gene abundance, and F:B ratio declined consistently in LMA and SMA (Supplementary Figures S5b, c, S6a). Stoichiometric ratios reflected altered nutrient demand: MBN:MBP and MBC:MBP decreased (Supplementary Figures S4a, b), while MBC:MBN increased (Supplementary Figure S4c), indicating a shift toward C limitation with enhanced P demand. In MA, all N addition levels promoted MBP accumulation, with the strongest response at N20 level (Supplementary Figure S3c). MBC and MBN followed the same decline-then-recovery pattern as other fractions but with more pronounced fluctuations (Supplementary Figures S3a, b). Notably, MA exhibited contrasting microbial community shifts: F:B ratio and *phoD* gene abundance increased with N addition (Supplementary Figures S5b, S6a), suggesting a dominance of fungi and functional bacteria. N addition enhanced ALP activity and PLFAs in all aggregates (Supplementary Figures S5a, S6c).

### Effects of plant, soil, and microbial properties on P fractions of soil aggregates

**Figure 2:**
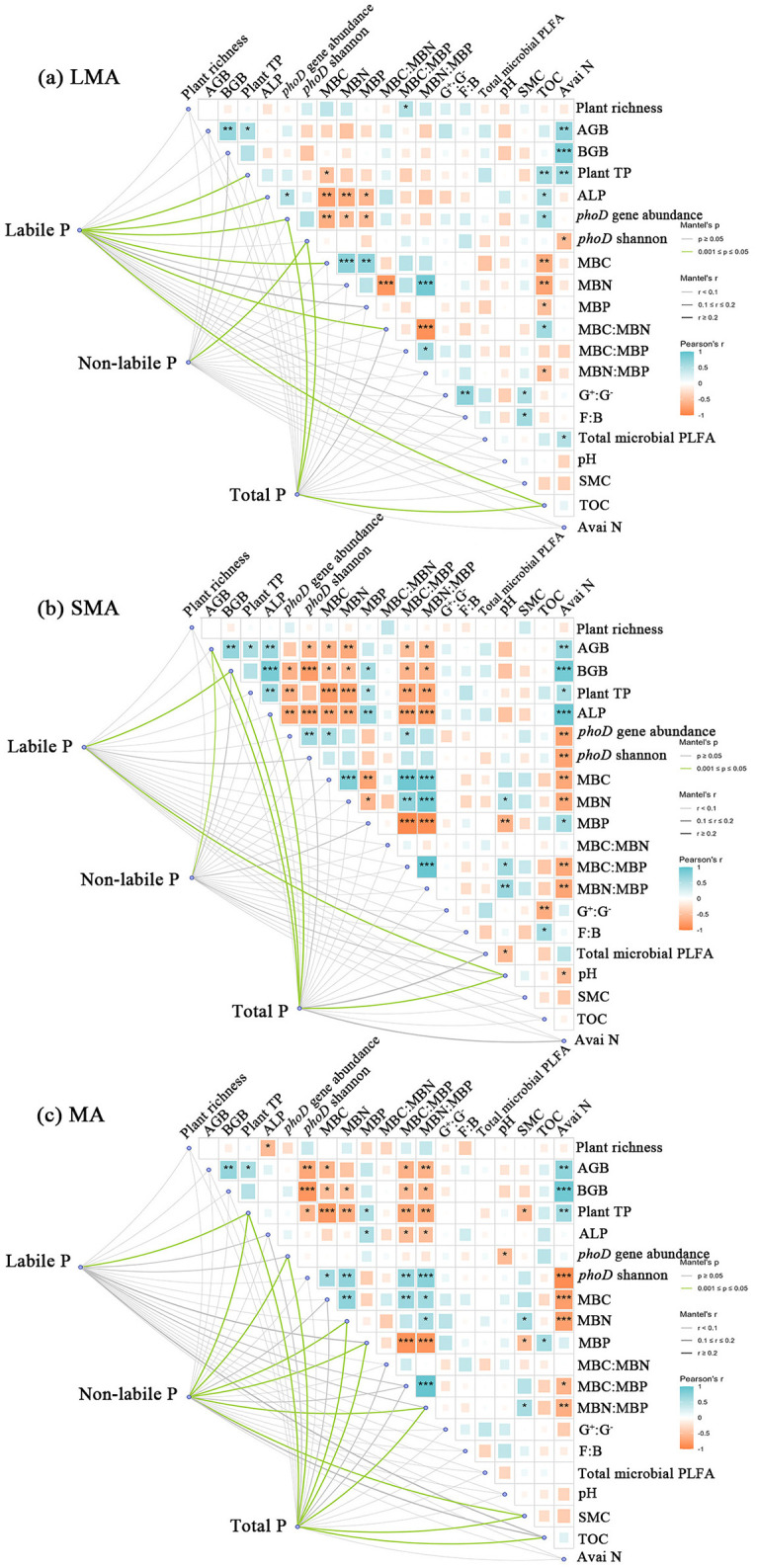
Partial Mantel test showing the relationship of plant, soil, and microbial properties with soil labile P and non-labile P in different size macroaggregates samples. The partial Mantel's r statistic is represented by line width, and the color of the line indicates the statistical significance (i.e., Mantel's p based on 999 permutations). Abbreviations of variables used in the partial Mantel test: Plant TP, plant total P; AGB, plant aboveground biomass; BGB, plant belowground biomass; TOC, total organic C; Avai N, available N; SMC, soil moisture content; ALP, alkaline phosphatase activity; *phoD* Shannon, soil *phoD* shannon diversity, *phoD* gene abundance, soil *phoD* gene abundance; G^+^:G^−^, the ratio of gram-positive bacteria to gram-negative bacteria; F:B, the ratio of fugal biomass to the bacteria biomass, The total PLFA, the total phospholipid fatty acid; MBC:MBP, the ratio of microbial biomass C to microbial biomass P; MBN:MBP, the ratio of microbial biomass N to microbial biomass P; MBC:MBN, the ratio of microbial biomass C to microbial biomass N. “*”, “**”, and “***” represent significance at *P* < 0.05, *P* < 0.01, and *P* < 0.001, respectively.

In the context of LMA, significant correlations were found between soil labile P and plant total P, *phoD* gene abundance, as well as organic C. Conversely, the Shannon index of *phoD* genes showed a significant association with soil non-labile P. Total P exhibited correlations with *phoD* gene abundance, *phoD* Shannon diversity, and soil organic C (Figure 2a). In SMA, soil labile P and non-labile P and total P were significantly correlated with all measured influencing factors (Figure 2b). Plant, soil, and microbial properties exerted a more dominant influence on P fractions in MA, demonstrating significantly stronger relationships than those observed in LMA. Specifically, soil labile P demonstrated significant correlations with ALP activity, MBP, MBC:MBP, and MBC:MBN, whereas soil non-labile P was linked to plant total P, *phoD* gene abundance, MBC, MBN, MBP, MBN:MBP, and SMC (Figure 2c).

**Figure 3:**
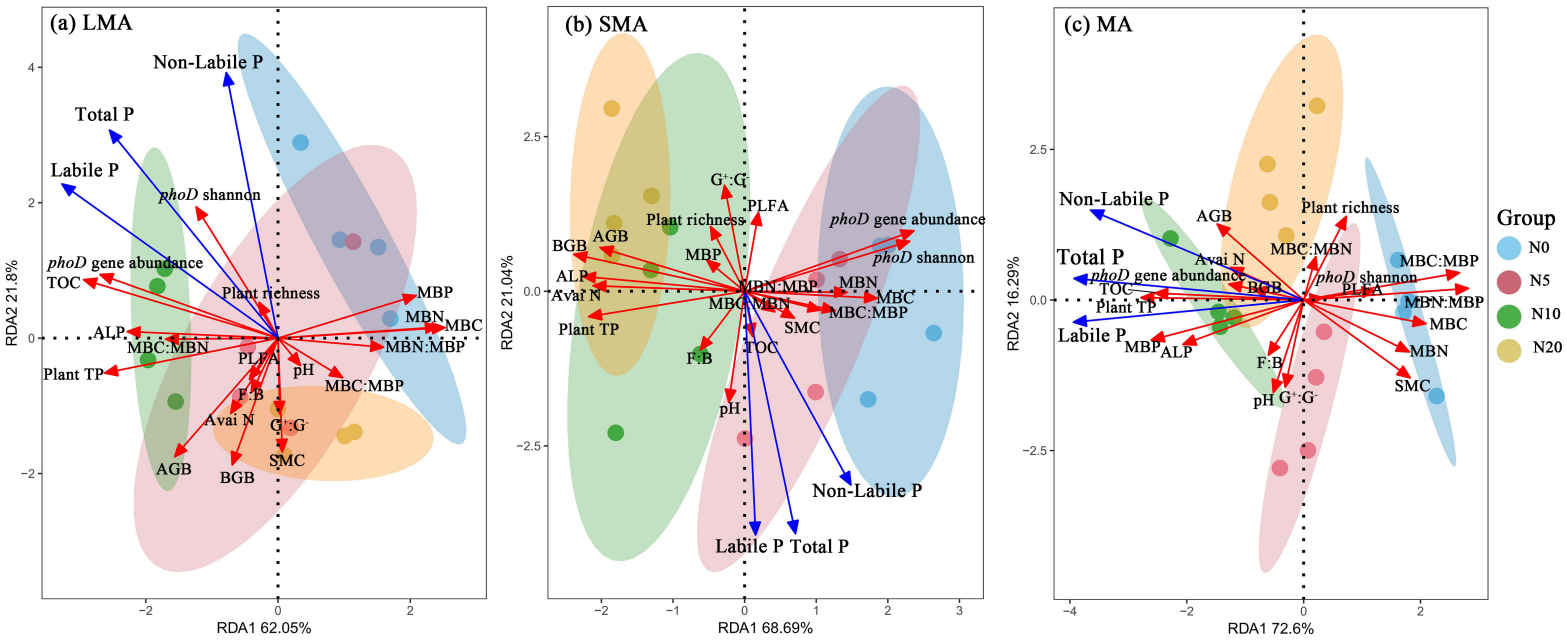
Redundancy analysis of plant, soil, and microbial properties, soil labile P and non-labile P in different size macroaggregates samples. Abbreviations of variables used in the redundancy analysis: Plant TP, plant total P; AGB, plant aboveground biomass; BGB, plant belowground biomass; TOC, total organic C; Avai N, available N; SMC, soil moisture content; ALP, alkaline phosphatase activity; *phoD* Shannon, soil *phoD* shannon diversity, *phoD* gene abundance, soil *phoD* gene abundance; G^+^:G^−^, the ratio of gram-positive bacteria to gram-negative bacteria; F:B, the ratio of fugal biomass to the bacteria biomass, The total PLFA, the total phospholipid fatty acid; MBC:MBP, the ratio of microbial biomass C to microbial biomass P; MBN:MBP, the ratio of microbial biomass N to microbial biomass P; MBC:MBN, the ratio of microbial biomass C to microbial biomass N.

**Figure 4:**
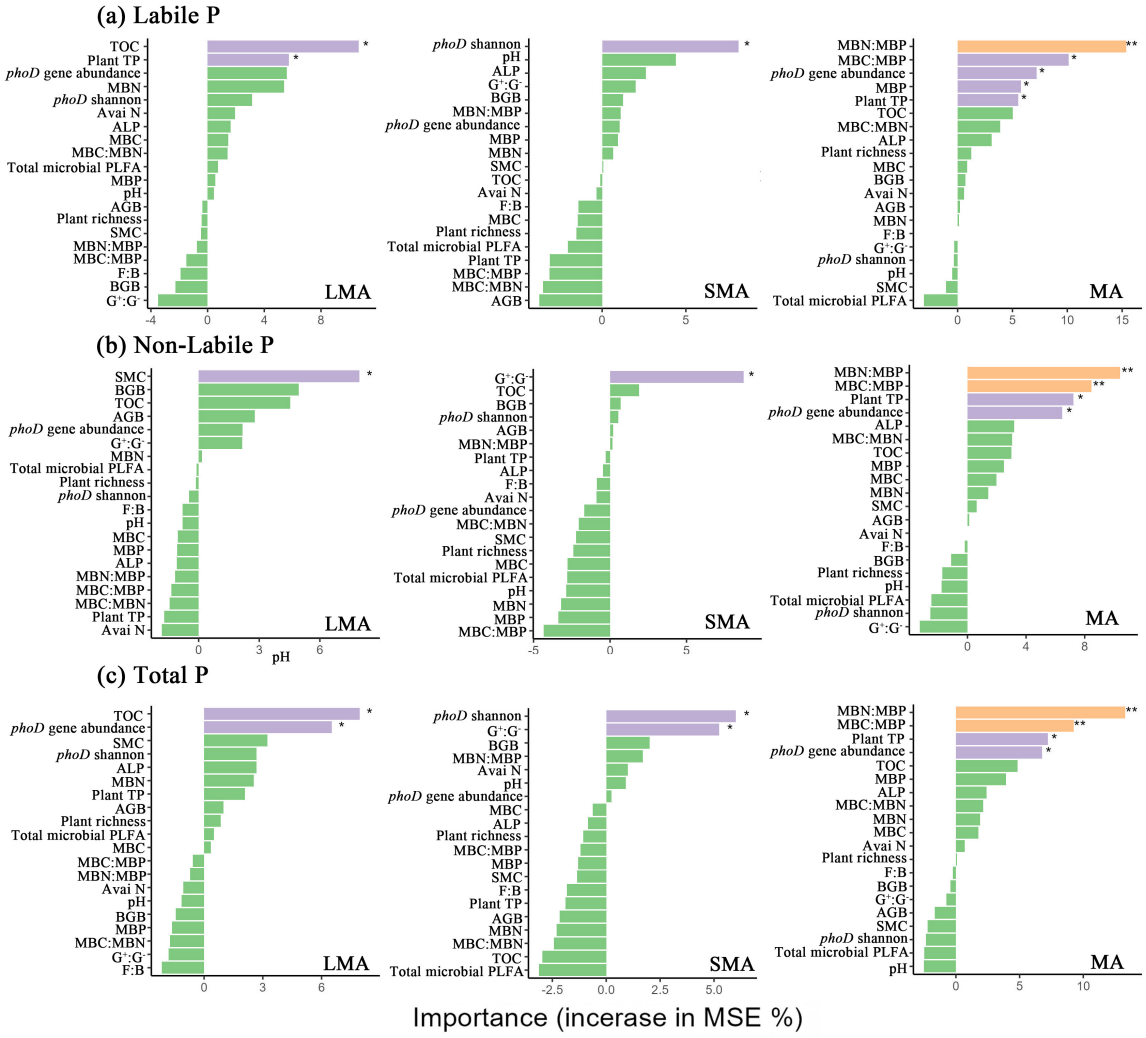
Results of mean square error (MSE, %) from a Random Forest aiming to identify the main driver of soil labile P, non-labile P and total P. Plant TP, plant total P; AGB, plant aboveground plant biomass; BGB, plant belowground plant biomass; TOC, total organic carbon; Avai N, available N; SMC, soil moisture content; *phoD* Shannon, soil *phoD* shannon diversity, *phoD* gene abundance, soil *phoD* gene abundance; MBC:MBP, ratio of microbial biomass carbon to microbial biomass P; MBN:MBP, ratio of microbial biomass N to microbial biomass P; MBC:MBN, ratio of microbial biomass carbon to microbial biomass N; ALP, alkaline phosphatase activity; MBP, microbial biomass P; MBC, microbial biomass carbon; MBN, microbial biomass N; F:B ratio, the ratio of fungi biomass to bacteria biomass. “*”, “**”, and “***” represent significance at *P* < 0.05, *P* < 0.01, and *P* < 0.001, respectively.

RDA revealed distinct drivers of soil P fractions across aggregate sizes (Figures 3a–c, Supplementary Table S1). In LMA, the model explained 83.85% of P fraction variation, with key drivers including plant richness, plant total P, *phoD* gene abundance, *phoD* Shannon diversity, ALP activity, MBC:MBN, and organic C (Figure 3a, Supplementary Table S1). For SMA, the model accounted for 89.73% of the variation, where primary influencing factors were the F:B ratio, soil pH, MBC:MBP, SMC, and organic C (Figure 3b, Supplementary Table S1). In MA, the model explained 88.89% of P fraction dynamics, with dominant drivers comprising AGB, BGB, plant total P, *phoD* gene abundance, MBP, ALP, soil organic C, and available N (Figure 3c, Supplementary Table S1). Random forest analysis revealed aggregate-specific predictors of soil P fractions (Figures 4a–c). In LMA, soil properties dominated predictions, with TOC being the strongest predictor for soil labile P and total P, followed by plant total P and *phoD* gene abundance. SMC was the primary predictor for soil non-labile P (Figure 4a). In SMA, microbial metrics were most influential. The Shannon diversity of the *phoD* gene explained most of the variance in soil labile P, whereas the G^+^:G^−^ ratio was the strongest predictor of soil non-labile P. For total P, *phoD* Shannon diversity G^+^:G^−^ ratio were critical (Figure 4b). In MA, microbial stoichiometric ratios governed predictions. MBC:MBP was the strongest predictor of variance across all P fractions, followed by plant total P, MBN:MBP, and *phoD* gene abundance (Figure 4c).

**Figure 5:**
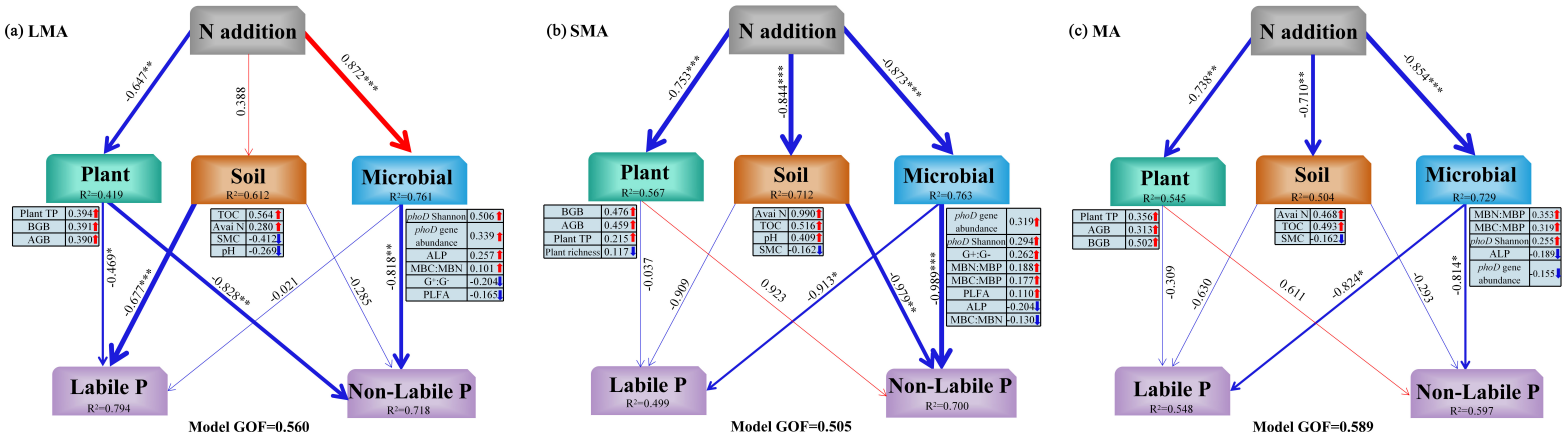
Partial least squares path modeling (PLS-PM) showing significant direct and indirect effects of plant, soil, and microbial properties on soil labile P and non-labile P in different size macroaggregates samples. Plant TP, plant total P; AGB, plant aboveground biomass; BGB, plant belowground biomass; TOC, total organic C; Avai N, available N; SMC, soil moisture content; ALP, alkaline phosphatase activity; *phoD* Shannon, soil *phoD* shannon diversity, *phoD* gene abundance, soil *phoD* gene abundance; G^+^:G^−^, the ratio of gram-positive bacteria to gram-negative bacteria; F:B, the ratio of fugal biomass to the bacteria biomass, The total PLFA, the total phospholipid fatty acid; MBC:MBP, the ratio of microbial biomass C to microbial biomass P; MBN:MBP, the ratio of microbial biomass N to microbial biomass P; MBC:MBN, the ratio of microbial biomass C to microbial biomass N. Single-headed arrows indicate causal relationships between variables. Numeric values on arrows represent standardized path coefficients. Red and blue arrows denote significant positive and negative effects, respectively. Values below variables indicate their explained variance (*R*^2^). GOF (goodness-of-fit) reflects the model reliability. The bar chart on the right shows the total effects of each factor on P fraction. “*”, “**”, and “***” represent significance at *P* < 0.05, *P* < 0.01, and *P* < 0.001, respectively.

**Figure 6:**
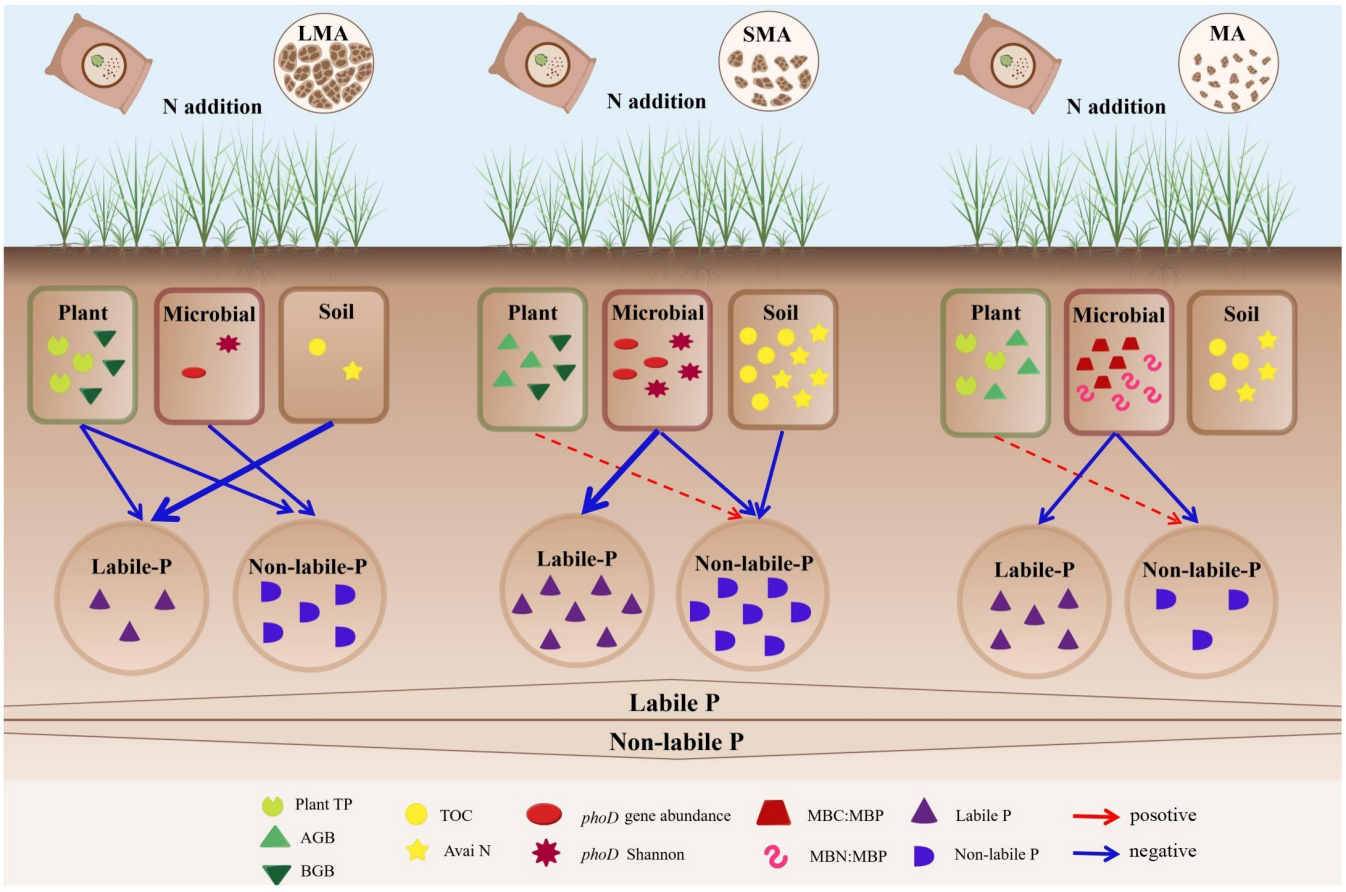
The conceptual diagram illustrates the driving factors that affect the contents of P components at different soil aggregate levels under the condition of N addition. The blue arrows indicate negative correlations, while the red arrows indicate positive correlations. The solid lines signify significant correlations, and the thicker the solid line is, the more significant the correlation is. The dashed lines indicate insignificant correlations. Plant TP, plant total P; AGB, plant aboveground plant biomass; BGB, plant belowground biomass; TOC, total organic C; Avai N, available N; *phoD* Shannon, soil *phoD* shannon diversity, *phoD* gene abundance, soil *phoD* gene abundance; MBC:MBP, ratio of microbial biomass carbon to microbial biomass P; MBN:MBP, ratio of microbial biomass N to microbial biomass P.

Our results of PLS-PM delineated direct and indirect pathways influencing P fractions (Figures 5a–c, 6). In LMA, soil labile P was directly regulated by soil properties and plant traits, while soil non-labile P responded to plant and microbial factors. Both pathways were indirectly mediated by N addition (Figures 5a, 6). In SMA, soil labile P was directly controlled by soil microbes, whereas soil non-labile P was driven by soil and microbial properties. N addition, indirectly affected both fractions through microbial community shifts (Figure 5b). In MA, microbial properties directly governed both soil labile P and non-labile P, with N addition indirectly modulating P dynamics via soil microbial stoichiometry (Figures 5c, 6).

## Discussion

### Effects of N addition on soil labile and non-labile P fractions across aggregates

Consistent with the hypothesis, the impact of N addition on soil labile P and non-labile P fractions depended on soil aggregates (Figure 1). Under N enrichment condition, the imbalance of soil N:P ratio induces P limitation for plants and microbes in soils (Walker and Syers, 1976). In response to the P limitation, plants and microbes modify their functional traits to efficiently acquire P, leading to competition between soil microbes and plants for P uptake (Wang et al., 2024; Jiang et al., 2024). We found that soil non-labile P in macroaggregates (i.e., LMA and SMA), and soil labile P in LMA under N5 and N10 additions decreased with N addition (Figure 1). The results of our study suggest that the intense P competition between plants and microbes is associated with a reduced capacity for microbial P immobilization (Eldridge et al., 2021). Conversely, N addition in MA increased both soil labile and non-labile P, possibly due to elevated soil organic C linked to N input (Zhao et al., 2025). Under N enrichment, plant roots secrete carboxylates to enhance soil organic C turnover and CO_2_ release, thereby acquiring more P (Luo et al., 2022). Soil organic C input drives soil P transformation via both chemical and biological mechanisms (Huang et al., 2022; Negassa et al., 2008). The accumulation of soil organic C could regulate P transformation through chemical processes such as dissolution and competitive adsorption, as well as via microbe-driven routes such as increased phosphatase release and the expansion of functional microbial community (Zhang Y. et al., 2023; Khan et al., 2019).

### N addition-driven response mechanisms of soil labile P

Soil aggregate composition is of great importance in regulating soil P dynamics (Pu et al., 2019; Chu et al., 2025). Microaggregates generally contain higher labile P than macroaggregates (Figure 1), with macroaggregates acting as a P supply pool and microaggregates as a storage pool due to their poor permeability and low microbial activity (Celik et al., 2010). At the aggregate level, a long-term N and water addition study showed that labile and residual P accumulate primarily in large macroaggregates, whereas Fe-/Al-bound P and organic P predominate in microaggregates, highlighting differential P stabilization across scales (Wang et al., 2010). These findings suggest that soil physical structure mediates P availability under changing N regimes. To improve the soil's ability to supply sufficient P for plants and microbes, we may need to protect the soil aggregates, especially the content of soil labile P in microaggregates, in a meadow steppe.

Our results show that *phoD*-harboring soil bacteria are sensitive to factors like organic C, with N addition promoting microbial decomposition of organic C and the release of occluded P (Torn et al., 1997). Microaggregates also retain more organic C and available N compared to macroaggregates (Supplementary Figure S2), enhancing their P retention capacity (Cui et al., 2023; Yan et al., 2023). However, in macroaggregates, N addition causes fluctuations in soil labile P due to their looser structure (Quesada et al., 2020). Our findings also indicate that N addition provides abundant N, fostering *phoD* gene abundance and microbial diversity in both aggregate types (Hu et al., 2020). Our study found that N addition provides abundant N for microbial life activities (Supplementary Figure S2a), while low organic C in LMA likely limits the C source required for *phoD* gene expression and ALP secretion (Nannipieri et al., 2011; Sun et al., 2023b). In large microaggregates (LMA), however, N addition may reduce available C sources for microbial activity, limiting *phoD* gene expression and ALP secretion, resulting in decreased abundance (Fan et al., 2024). When C and N sources are insufficient, microbes prioritize basic metabolism over ALP synthesis, thus reducing activity and restricting organic P mineralization (Tan et al., 2013).

### Regulatory mechanisms of soil non-labile P under N addition conditions

In contrast to macroaggregates, where soil non-labile P decreases with N addition (Figure 6), microaggregates show an increase in non-labile P with N enrichment. This is attributed to N-induced soil acidification, which enhances the solubility of metal oxides, altering their adsorption capacity for P (Supplementary Figure S2b) (Namuhan et al., 2024). N addition also influences ALP secretion, thereby affecting P transformation processes (Yu et al., 2024). As plant biomass increases with N addition (Supplementary Figure S1), roots secrete organic acids, acidifying the rhizosphere and enhancing P availability (Zhi et al., 2025). Additionally, N addition increases soil microbial biomass P (MBP) while decreasing microbial biomass C and N (Supplementary Figure S3), reflecting reduced microbial demand for P in microaggregates (Wang et al., 2023; Li et al., 2025). Our findings are consistent with previous studies, showing that N addition promotes P cycling in soils by increasing MBP (Chen et al., 2025).

N enrichment disrupts the soil C:N:P balance, prompting microbial activity changes and altering P dynamics (Wang et al., 2024; Zhu et al., 2023; Wei et al., 2019). Microbial growth follows the Redfield ratio (Redfield, 1958). Microbes in microaggregates enhance P uptake to maintain their C:N:P stoichiometry, converting labile inorganic P into organic P, thus increasing soil MBP (Supplementary Figure S3). Organic P in particulate forms decomposes over time, contributing to non-labile P in microaggregates (Peng et al., 2022; Fan et al., 2024).

N addition also decreases the diversity of *phoD*-harboring bacteria in microaggregates (Supplementary Figure S5). This promotes ALP activity, but the rate of biological P assimilation surpasses mineralization, resulting in reduced labile P and increased non-labile P accumulation (Liu et al., 2024). Microbial-produced extracellular polymers enhance aggregate stability, but their compact structure limits substance diffusion (Olagoke et al., 2022; Shi et al., 2024). Consequently, ALP concentration increases locally, improving catalytic efficiency but hindering P release due to adsorption by metal oxides or precipitation (Xu et al., 2022). Future studies using stable isotope tracing could differentiate the contributions of biological and chemical processes to non-labile P accumulation (Chen et al., 2024a,b). This study is limited to a single experimental site; therefore, future work should involve multi-site experiments at a global scale to further validate the conclusions.

## Conclusion

N enrichment effects on soil P fractions vary significantly among aggregate levels, as demonstrated in this study. In all N treatments, non-labile P in macroaggregates consistently decreased, while it increased in microaggregates. Soil non-labile and labile P in microaggregates showed a decreasing trend across all N addition levels. In macroaggregates, soil P was primarily controlled by soil properties and microbial characteristics, such as soil C, N, and *phoD*-harboring bacterial diversity. In microaggregates, microbial stoichiometry played a crucial role in driving the dynamics of soil P in response to N addition. Our findings highlight that soil aggregates regulate microbial functional traits' impact on P cycling and supply under N deposition. The differential response patterns across aggregate levels underscore the complexity of soil P cycling, integrating plant–microbe–soil feedbacks and stoichiometric controls at fine spatial scales. This study provides new insights into the role of soil functional microbes and stoichiometry, emphasizing the need for attention to soil P management in the context of global change.

## Data Availability

The original contributions presented in the study are publicly available. This data can be found here: NCBI, accession number PRJNA1345169.
